# Navigating Fertility Preservation Options in Gynecological Cancers: A Comprehensive Review

**DOI:** 10.3390/cancers16122214

**Published:** 2024-06-13

**Authors:** Elena Chitoran, Vlad Rotaru, Madalina-Nicoleta Mitroiu, Cristiana-Elena Durdu, Roxana-Elena Bohiltea, Sinziana-Octavia Ionescu, Aisa Gelal, Ciprian Cirimbei, Mihnea Alecu, Laurentiu Simion

**Affiliations:** 1 School of Medicine, “Carol Davila” University of Medicine and Pharmacy, 050474 Bucharest, Romania; chitoran.elena@gmail.com (E.C.); sinzianaionescu30@gmail.com (S.-O.I.);; 2 General Surgery and Surgical Oncology Department I, Bucharest Institute of Oncology “Al. Trestioreanu”, 022328 Bucharest, Romania; 3 Obstetrics and Gynecology Department, “Filantropia” Clinical Hospital, 011132 Bucharest, Romania

**Keywords:** fertility preservation, gynecological cancers, assisted reproductive technologies, conservative surgery, cancer survivorship, quality of life, onco-fertility

## Abstract

**Simple Summary:**

Gynecological cancers affect many women of reproductive age, necessitating the development of fertility preservation methods to fulfill family planning. Consequently, providing treatment options that preserve fertility in women diagnosed with gynecological cancers has become a crucial component of care for survivors. This leads to improved quality of life, allowing survivors to become mothers even in the seemingly adverse circumstances posed by cancers. However, although there are guidelines on fertility preservation in the context of neoplasms, physicians do not routinely consider it and do not discuss these options with their patients. It is important for patients to be informed about the available options for fertility preservation and to be encouraged to make informed decisions in collaboration with their medical team. Standardized guideline recommendations for onco-fertility should be considered in the future. We explore possible methods that can be employed for obtaining a pregnancy after gynecologic cancer treatment, including more exotic ones like uterine transplant.

**Abstract:**

(1) Background: Currently, an increasing number of women postpone pregnancy beyond the age of 35. Gynecological cancers affect a significant proportion of women of reproductive age, necessitating the development of fertility preservation methods to fulfill family planning. Consequently, providing treatment options that preserve fertility in women diagnosed with gynecological cancers has become a crucial component of care for survivors. (2) Methods: We conducted an extensive search of relevant scientific publications in PubMed and Embase databases and performed a narrative review, including high-quality peer-reviewed research on fertility after being treated for gynecologic cancers, reporting pregnancy rates, birth rates, and pregnancy outcomes in cancer survivors as well as therapeutic options which partially preserve fertility and methods for obtaining a pregnancy in survivors. (3) Discussion: The medicine practiced today is focused on both treating the neoplasm and preserving the quality of life of the patients, with fertility preservation being an important element of this quality. This leads to an improved quality of life, allowing these women to become mothers even in the seemingly adverse circumstances posed by such a pathology. However, although there are guidelines on female fertility preservation in the context of neoplasms, an analysis shows that physicians do not routinely consider it and do not discuss these options with their patients. (4) Conclusions: Advancements in medicine have led to a better understanding and management of gynecological neoplasms, resulting in increased survival rates. Once the battle against these neoplasms is won, the issue of preserving the quality of life for these women arises, with fertility preservation being an important aspect for women who have not yet fulfilled their family planning desires at the time of diagnosis. It is important for patients to be informed about the available options for fertility preservation and to be encouraged to make informed decisions in collaboration with their medical team. Standardized recommendations for onco-fertility into guidelines should be taken into consideration in the future.

## 1. Introduction

Even though an ever-increasing number of young women diagnosed with cancer will survive this diagnosis, most will face long-term repercussions, one of which is infertility [1]. Thus, the medicine practiced today is focused on both treating the neoplasm and preserving the quality of life of the patients, with fertility preservation being an important element of this quality. Fertility preservation in the context of female genital neoplasms encompasses all surgical and non-surgical methods of preserving female reproductive capacity and represents the definition of a new medical branch called onco-fertility [2]. This leads to an improved quality of life (QoL), allowing these women to become mothers even in the seemingly insurmountable circumstances posed by such a pathology. Although there are guidelines on female fertility preservation in the context of neoplasms, most physicians do not routinely consider it and do not discuss these options with their patients [1]. Given that gynecological neoplastic pathologies can occur in young, reproductive-aged women, the issue of fertility preservation through both surgical and non-surgical management has been raised. The evolution of oncological gynecology has led to a better understanding and management of neoplastic pathologies. This has directed specialists’ attention towards improving the QoL of patients after the completion of cancer treatment, not just the treatment itself. Considering the increased average age of conception in modern women worldwide, from 26.7 years in 2012 to 28.4 years in 2021 according to UNECE (United Nations Economic Commission for Europe) data, with countries like Italy and Spain having an extreme average conception age (31.6 years in 2021), the birth rate is hindered even outside of neoplastic pathology. Given that gynecologic cancers predominantly occur in women over 30, there is concern about the existence of two negative prognostic factors when it comes to fertility, namely neoplastic pathology and advanced age. The treatment of gynecologic cancers affect fertility by variate mechanisms: absolute uterine factor infertility (AUFI), reduced ovarian reserve, radio-chemotherapy effects on internal genital organs, and delayed procreation due to the effects of cancer treatment. It is crucial to ensure that all young patients are given the chance to become mothers, which is why counseling them before starting neoplastic treatment within a multidisciplinary team is essential to practice medicine to the highest standards [3]. Adequate treatment options should be considered for each patient, taking into account all other factors that could influence fertility in these patients like the presence of chronic endometriosis or even the genital tract microbiome [4,5]. The methods of fertility preservation for cervical, endometrial, ovarian, and breast cancers will be reviewed in the following sections. The aim of this study is to evaluate the current therapeutic option that ensures both good control of neoplastic disease and adequate fertility preservation in gynecologic malignancies. Also, we have reviewed all techniques that can be used for obtaining pregnancies in gynecologic cancer survivors. The present study evaluates fertility possibilities after cervical, endometrial, ovarian, and breast cancers.

## 2. Methods

For the purpose of this study, we conducted an extensive search of relevant scientific publications in PubMed and Embase databases and performed a narrative review. All peer-reviewed research and conference papers on the subject of fertility after being treated for gynecologic cancers were considered eligible for inclusion. We included articles reporting pregnancy and birth rates as well as pregnancy outcomes in cancer survivors. Articles about the therapeutic options that preserve fertility, at least in part, and methods for obtaining a pregnancy in such patients were also included.

With regard to publication type, all records reporting results from randomized controlled trials, cohort studies, cross-sectional studies, reviews, and meta-analyses were considered eligible for this narrative review. In contrast, case reports and case series were excluded due to low evidence levels. Also, records were excluded if they did not have available full text, if they reported results from animal studies, and if they were published in a language other than English, Spanish, or Romanian. Records pertaining to other cancers besides gynecologic cancers were also excluded.

The initial search was performed using Mesh terms and keywords (describing gynecologic malignancies, survivorship status, and fertility) connected by appropriate Boolean operators. The following syntax components were used for database searching:

1. Pregnancy OR birth OR reproduct* OR fertility preservation OR fertility sparing OR onco-fertility OR onco-fertility.
2. Gynecologic cancer OR cervical cancer OR endometrial cancer OR ovarian cancer OR breast cancer.
3. Cancer survivor*.
4. Filters: full-text available, research pertaining to humans, publication type as described above.

Filters applied to database searches, particularly “full-text available”, may in fact result in not retrieving some relevant articles, and the syntax used may always be improved by the addition of other (near-)synonyms, but for the purpose of this narrative review, we considered this search strategy as sufficient. However, we admit as a potential limitation of the study the fact that our search strategy may have not included all relevant sources available. Additional relevant records were identified via reference screening.

All retrieved records were electronically screened and duplications were removed. Subsequently, 2 independent reviewers screened the records by titles and abstracts, and excluded records that did not meet our inclusion criteria. After this process relevant records were selected for full-text retrieval. The texts were assessed by two independent reviewers; discrepancies were solved via group discussion and a senior reviewer’s opinion was taken into consideration in case of disagreement.

This narrative review contains a qualitative but not a quantitative summary of findings, because of the expected high heterogeneity of articles included. Also, most studies report outcomes for very small numbers of patients which may not allow for a proper statistical analysis.

## 3. Results

**Figure 1:**
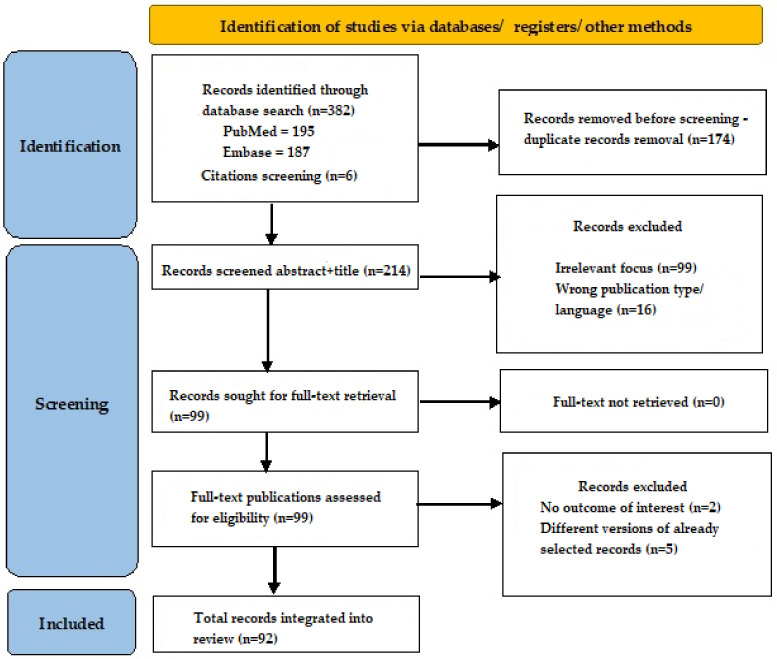
Search flow diagram.

After this search, we obtained 195 results in PubMed and 187 results in Embase. An additional six records were identified via reference screening. All records were written between 1997 and 2024. After duplicate record removal, we obtained 214 records and then screened the titles and abstracts for eligibility; 115 records were excluded due to irrelevant focus or not meeting inclusion criteria. Full-text articles were retrieved for 99 records, and out of those, 92 were integrated into our review according to relevance to the discussed subject (Figure 1).

## 4. Discussion

As a general rule, all patients diagnosed with gynecologic malignancies should undergo comprehensive preoperative staging (clinical, imaging, and even surgical). All patients wishing to keep their reproductive potential should be counseled and informed on the risks associated with this option (potential worse oncologic outcomes and complications associated with assisted reproduction technologies and possible side-effects of cancer treatments on gestation and birth—including teratogenic effects). Patients need to be aware of the realty of gestation after cancer treatment, which may be difficult to achieve and even more difficult to carry to term. Prior to fertility-sparing surgery, patients should be evaluated by an endocrinologist and should undergo extensive genetic testing to identify carriers of the BRCA mutation or other hereditary cancer genes related to gynecologic cancers, and if such mutations are present fertility-sparing surgery should be contraindicated. Carrying such genes should be considered as the main features to define a candidate for conservative management of disease. In this light, it becomes clear why the decision to preserve fertility should be made by the patient together with her medical team after extensive evaluation of all factors described, and after ensuring she has fully understood the implications of such a decision.

### 4.1. Ovarian Cancer

Ovarian cancer is known for a low percentage (40%) of early-stage diagnosis eligible for surgical fertility preservation. According to the National Comprehensive Cancer Network (NCCN) and the European Society for Medical Oncology (ESMO), fertility preservation surgery can be considered and performed only in patients who wish to retain their reproductive potential and have early-stage epithelial tumors (stages IA to IC) or low-risk tumors such as low-malignant potential (LMP) lesions, borderline tumors, germinal cell tumors, or sex-cord malignant tumors. Young patients with early-stage ovarian dysgerminomas or pure immature teratomas are generally the best candidates for conservative management, since these types of tumors are associated with high 5-year survival rates (both global and disease-free survival, reaching up to 98.7% and 94.6%, respectively, according to some studies [6]) and low recurrence rates (ranging from 3.9% in patients who received adjuvant chemotherapy to 8.3% in patients that did not) [6]. Recent studies have shown that early-stage clear cell ovarian cancers and even more advanced borderline tumors, when treated with fertility-sparing surgery, are not associated with worse outcomes than conventional radical procedures [7,8].

Unlike the radical approach (which involves total hysterectomy with bilateral salpingo-oophorectomy, omentectomy, peritoneal lavage, and tumor staging through pelvic and para-aortic lymphadenectomy and multiple peritoneal biopsies), fertility preservation surgery includes unilateral salpingo-oophorectomy and tumor staging through pelvic and para-aortic lymphadenectomy, peritoneal biopsies, and omentectomy, preserving the unaffected ovary and uterus. A complete surgical staging still needs to be conducted in patients with apparent incipient disease (upon clinical and imaging evaluation), due to the fact that around 30% of such patients are upstaged after surgical exploration [9,10,11,12,13,14,15]. Complete surgical staging may be omitted in pediatric patients with apparent early-stage germinal cell malignant tumors since incomplete staging does not seem to be associated with poorer survival outcomes [16]. However, for adults presenting with apparent early-stage germinal cell malignant tumors, a complete surgical staging is recommended; a number of retrospective studies suggest that there seems to be a significant association between increased risk of recurrence and incomplete surgical staging [17,18].

Adjuvant chemotherapy can be administered after radical or fertility-sparing surgery, depending on the case. Regarding the spared ovary, a biopsy can be considered depending on the case, but it should be noted that this gesture may lead to ovarian adhesions over time and could adversely affect the ovarian reserve [2]. Several studies support the surgical preservation of the contralateral ovary in stages IA and IC of the disease, observing a recurrence of under 5% in the spared ovary [2]. Under these conditions, the 5-year survival rate for ovarian cancers is over 80%, and the success rate of conception ranges from 60% to 100% [19]. As for borderline tumors, the 10-year recurrence-free survival rate ranges from 77% in stage IV to 99% in stage I [19]. In a study conducted over 12 years, including 154 women aged 18 to 45 out of a total of 1618 women diagnosed with stages IA and IC ovarian cancer reported in the California Cancer Registry, it was concluded that women who conceived at least 3 months after undergoing fertility preservation surgery did not have an increased risk of maternal and neonatal mortality, nor a higher risk of premature birth or small-for-gestational-age infants [20]. Thus, the existing literature considers conception after an ovarian cancer diagnosis approached by fertility-sparing surgery as safe for both the mother and the fetus.

At this moment, the impact of chemotherapy on ovarian function and ulterior conception rates is insufficiently known [21]. Regarding women undergoing adjuvant chemotherapy, it is worth mentioning how chemotherapy delays the time of conception by 1–2 years. Chemotherapy-induced prolonged immunosuppression [22,23] can impair oocytes and lead to complications such as miscarriage, premature birth, or small-for-gestational-age infants. In this regard, it has been observed that combining chemotherapy with a synthetic analog of luteinizing hormone called goserelin increases the pregnancy rate compared to chemotherapy alone [24]. Also, using a gonadotropin-releasing hormone [25,26] (GnRH) agonist seems to offer protection against premature ovarian insufficiency, but at this moment, we do not have enough data to support this, with several studies stating that there is no actual benefit in protecting fertility [27]. This combination therapy aids in preserving ovarian function, a distinction that must be made from the term fertility preservation. Currently, GnRH agonist therapy is the only recommended approach for preventing premature ovarian insufficiency. The prospective study PREFER highlighted how GnRH agonist therapy is embraced by a significant percentage of patients, with over 90% opting for this treatment during chemotherapy. The aim of this therapy was to prevent the consequences of a dramatic decrease in estrogen levels and all its associated effects. However, it should be noted that at this moment, the mechanism by which this suppression therapy protects ovarian function is not fully understood. Further research is needed to fully comprehend these mechanisms [28].

The long-term teratogenic effects of chemotherapy on children conceived several years after chemotherapy treatment have not been demonstrated. As for pregnancies obtained shortly after completing chemotherapy, both an increase in abortion rate and the appearance of congenital malformations have been recorded. Thus, in the case of a pregnancy occurring shortly after completing chemotherapy treatment, careful monitoring of congenital defects and chromosomal aberrations is recommended [29]. The time of conception should be approximately two years after completing cancer treatment [30]. In the case of fertility-sparing surgery followed by assisted human reproduction, there are insufficient studies to attest to the long-term safety of this procedure. Additional studies are needed for firm conclusions [28].

Regarding the need for radiotherapy (which is not recommended by current guidelines in ovarian tumors, but may be considered in selected cases), to avoid its teratogenic effect on the highly radiosensitive ovarian tissue, surgical transpositioning of the ovaries can be performed, moving them to an area outside the radiation field. The preferred approach for ovarian transposition is laparoscopy, but it can also be achieved through laparotomy [31]. Additionally, in vivo or in vitro oocyte or embryo cryopreservation represents a safe alternative for fertility preservation as well as ovarian tissue preservation. Later, it was found that in vivo ovarian puncture in order to collect the oocytes carries the risk of peritoneal seeding, and it was found that this procedure can also be performed after surgical annex resection to avoid that risk [32]. The age cut-off for the previously mentioned methods (oocyte preservation, embryo preservation, ovarian tissue preservation) is 36–40 years [33].

In more advanced ovarian tumors (stage II or III), fertility-sparing surgery is unconventional and is not recommended. In such cases, the focus of surgery should be a maximal cytoreductive effort, which leads to absolute uterine factor infertility (AUFI) and absolute ovarian insufficiency. But even in such cases, prior to cancer therapy, the patient may opt for oocyte or embryo cryopreservation, which may be used later to fulfill family planning needs through the use of surrogacy. More exotic options such as uterine transplant may be considered, but at this point in time, the technology is still under evaluation and needs further proof.

### 4.2. Cervical Cancer

Cervical cancer is the fourth most common cancer among women and the fourth leading cause of cancer mortality among women worldwide [34]. Among patients diagnosed with cervical cancer, 43% are under 45 years old and 20–28% are under 40 years old [35,36,37]. The current treatment for cervical cancer depends on tumoral staging [38,39] and consists of simple or radical hysterectomy with regional lymphadenectomy, chemotherapy, or radiotherapy, these methods most often leading to the irreversible loss of the ability to bear a child.

The development of less invasive surgical techniques to preserve fertility currently offers fertile women with cervical cancer the possibility of obtaining a pregnancy [40]. The first procedure of this type was described by Dargent and consists of radical vaginal trachelectomy with laparoscopic pelvic lymphadenectomy, this technique being indicated in the case of tumors <2 cm [41]. Radical trachelectomy involves the complete detachment of the vagina from the uterus by resection of the cervix, the upper third of the vagina, and the surrounding parameters [42]. This procedure is associated with a pregnancy rate of 24%, a recurrence rate of 4.2%, and a mortality rate of 2.9% [43]. The recurrence rate in the case of radical abdominal trachelectomy is 1.6%, while the mortality rate is 0.5% [44,45]. Lintner et al. demonstrated a 5-year disease-free survival rate similar to radical hysterectomy [46]. A systematic review demonstrated that 59.3% of patients who underwent a radical abdominal trachelectomy were able to achieve a pregnancy [33]. Abdominal radical trachelectomy has been associated with obstetric complications such as abortion in the first or second trimester, premature birth, cerclage erosion, varicose veins at the level of the uterovaginal anastomosis, chorioamnionitis, premature rupture of the membranes, and increased need for cesarean sections [47].

Currently, the data from randomized controlled trials regarding recurrence and pregnancy rates after different types of trachelectomy (vaginal, abdominal, and minimally invasive) are limited, but the numerous retrospective and prospective studies suggest similar results, with the existing alternatives being feasible options [44]. While minimally invasive trachelectomy is associated with better visualization of the fallopian tubes, less blood loss, shorter hospitalization, and a better cosmetic result of postoperative scars, it is also time-consuming, adds supplementary costs, requires a long learning curve, and is dependent on the skills of the operator. Additionally, it can be limited by the body mass index of the patient [46,47]. Although abdominal trachelectomy allows for better resection of large tumor masses, it has been suggested that more extensive resection could be associated with a poorer obstetrical prognosis. Therefore, controlled randomized studies involving a large number of patients are needed to establish the superiority of one technique over the other [46].

Although trachelectomy has been shown to be safe and has results similar to standard surgical procedures, it is associated with certain complications. By affecting the inferior hypogastric plexus, this technique can lead to urinary or digestive pathologies [47]. Furthermore, suturing the isthmic portion of the uterus to the vagina is associated with impaired uterine continence, increased rates of preterm births (20–30%), and a rise in the number of abortions in the first and second trimesters (16–20% and 8–10%) [48,49]. Therefore, new surgical methods have been developed to decrease the complication rate and improve the pregnancy rate.

One of these methods is represented by conization with laparoscopic parametrial tissue excision and pelvic lymphadenectomy. This technique can be used in the case of cervical cancer stage IA1 FIGO patients [50] (FIGO—classification of the International Federation of Gynecology and Obstetrics). To ensure the oncological safety of the procedure, a safety margin of at least 8 mm must be ensured [51]. Otherwise, re-conization or trachelectomy may become necessary [52]. A meta-analysis comparing different types of conization (cold-knife conization, LEEP (loop electrosurgical excision procedure), or LLETZ (large loop excision of the transformation zone)) demonstrated that there is no significant statistical difference regarding recurrence rate, cervical stenosis, secondary hemorrhage, positive margins, and residual disease rate [53].

In the case of patients with stage IA2 FIGO and IB1 FIGO with tumors <2 cm, the recommended treatment is radical vaginal trachelectomy, but some centers have also used conization in combination with lymphadenectomy [54,55]. For patients with IB FIGO stage with tumor size >2 cm, alternatives to Dargent’s procedure are used in selected cases, with the techniques being represented by radical abdominal trachelectomy or minimally invasive radical trachelectomy (laparoscopic or robotically assisted), which are considered suitable for tumors >2 cm as they ensure a better resection of the parameters [56].

For more advanced stages (IB2, IIA1), the specialized literature describes the use of neoadjuvant chemotherapy (NAC) followed by fertility preservation surgery (radical trachelectomy, simple trachelectomy, cold-knife conization, or laser conization) [57].

Pregnancy rates in cancer survivors are lower when compared to the general population, and cervical cancer is associated with the lowest chance of ulterior pregnancies [58]. However, in some studies, around 55% of early-stage cervical cancer treated by fertility-sparing surgery became pregnant and 70% of them delivered a live child [57]. Neoadjuvant chemotherapy seems to have a favorable effect on pregnancy and live birth rates [57]. Some studies have suggested that radical vaginal and simple trachelectomy are associated with higher rates of conception than other fertility-sparing options [59].

Fertility-sparing management of cervical neoplasia does not necessarily mean that the patient will be able to conceive. Infertility prevalence after abdominal radical trachelectomy was reported in between 33% and 58% of women who desired to conceive after the procedure [60,61]. Fertility-sparing procedures can result in infertility subsequent to specific complications like cervical stenosis (following radical trachelectomy [59,60,61,62]), which may be difficult if not impossible to overcome [62]. Severe forms may be overcome by using assisted reproductive technologies [63,64]. Similar, but in the opposite way, loop electro-excision or cold-knife conization can lead to cervical incompetence and an increased chance of preterm births through progression during pregnancy [65] and a higher chance of intrauterine infections [66].

It should be noted, however, that fertility-sparing surgery in advanced cervical cancer is unconventional and not recommended by guidelines. In such cases, like in ovarian cancer, patients should opt for pretreatment oocyte or embryo cryopreservation and surrogacy.

### 4.3. Endometrial Cancer

Endometrial cancer is the sixth most common cancer in women, with 417,000 new cases and 97,000 deaths worldwide in 2020 [34]. This type of cancer usually appears in the postmenopausal period, the average age of diagnosis being 65 years. However, 4% of patients are under 40 years old, and 70% of these younger patients do not have children at the time of diagnosis [67]. These patients usually have a good prognosis because more than 95% of these tumors are of the endometrioid type, well differentiated and limited to the endometrium or superficial myometrium, most being diagnosed in stage IA FIGO [68]. Both the incidence and mortality rates of endometrial cancer have been on the rise, accompanied by a noticeable decline in the age at which the disease typically manifests across various types of endometrial pathologies [69]. In women diagnosed with endometrial cancer, the treatment options consist of surgical and non-surgical methods depending on tumor stage. In terms of surgical treatment, this consists of total hysterectomy using the classical method, or minimally invasive robotic or laparoscopic surgery, with or without bilateral adnexectomy, and if necessary, pelvic and para-aortic lymphadenectomy [70].

Fertile patients diagnosed with endometrial cancer who wish to become pregnant could benefit from conservative treatments under certain conditions. The criteria that must be met for conservative treatment to preserve fertility are represented by a histopathological diagnosis of well-differentiated endometrioid endometrial carcinoma, confirmed by an experienced pathologist; the tumor must be limited to the endometrium on MRI or transvaginal ultrasound examination; there should be no imaging suspicion of metastases and no contraindications to the treatment or becoming pregnant. Conservative treatment is not recommended for patients with other types of tumors such as clear cell carcinoma, serous carcinoma, choriocarcinoma, sarcoma, and undifferentiated tumors [71,72].

In 2013, The Cancer Genome Atlas (TCGA) Research Network provided the medical community with a new prognostic classification of endometrial cancer based on the tumor’s molecular biology [73] in four categories: POLE ultra-mutated, microsatellite instability hypermutated, low-copy-number tumor, and high-copy-number tumor. Each group has specific genetic characteristic with distinct mutations and are associated with different prognosis. The first category—POLE ultra-mutated tumors—has the most favorable prognosis and is associated with a longer progression-free survival. It is usually correlated with the endometrioid histology type [74]. Microsatellite instability hypermutated or mismatch repair-deficient tumors are characterized by an intermediate prognosis and are also associated with the endometrioid subtype [74]. Similarly, the third category is also characterized by an intermediate prognosis and associated with endometrioid histology. The last category—high-copy-number tumors—is associated with unfavorable prognosis and serous histology. Molecular and genetic markers can be determined before planned treatment together with immunohistochemical examinations on tumor biopsy samples, and play a role in predicting responses to treatment [75,76], in risk-stratifying [77], and, possibly, in better selecting patients that would benefit the most from fertility-sparing management. The integrated clinical, pathological, and molecular tumor characteristics for each category were summarized in a consensus published in 2021 [78].

Criteria for attempting fertility-sparing surgery or non-surgical methods are summarized in Table 1 for both endometrial cancer and other gynecologic malignancies.

Most endometrial cancers (80–90%) are type I and are estrogen-associated, with the development of this type of cancer being closely related to the uncontrolled action of estrogen on the endometrium [70]. Progesterone is a steroid hormone that counteracts the carcinogenic action of estrogen on the endometrium, acting by activating the enzymatic substances that participate in the metabolism of estrogen and by downregulating estrogen receptors. In addition, progesterone has an antitumoral effect by regulating the cell cycle through cyclin-dependent kinases and modulating the activity of oncogenes [79].

Considering these mechanisms, the current non-surgical conservative treatment consists of oral administration of progesterone, either megestrol acetate (160–320 mg/day) or medroxyprogesterone acetate (400–600 mg/day). These types of progesterone are administered orally daily, and in the case of medroxyprogesterone acetate, it can also be administered intramuscularly twice a week [80]. Regarding the remission rate, megestrol acetate has better results than medroxyprogesterone acetate, which can be explained by a higher bioavailability [81].

Another proposed therapeutic strategy is represented by the levonorgestrel-releasing intrauterine system (LNG-IUS), in combination with oral progestins with or without gonadotropin-releasing hormone (GnRH) analogs [82,83]. LNG-IUS in combination with oral progesterone and GnRH has better results in terms of remission, recurrence, and relapse rate, compared to LNG-IUS alone [84,85].

Other therapeutic substances have been proposed as fertility preservation therapeutic options in patients with endometrial cancer, but there are little to no studies to attest their effectiveness. These methods are represented by the administration of natural progesterone, norethisterone acetate, hydroxyprogesterone caproate, combined oral contraceptives, and aromatase inhibitors (anastrozole, letrozole) [86,87].

Another fertility-sparing approach consists of hysteroscopic resection of the tumor followed by oral or intrauterine administration of progesterone [78,88,89,90]. Patients treated by this method have a regression, recurrence, and pregnancy rate of 97.4%, 3.6%, and 47.4%, respectively [91]. Hysteroscopic resection not only ensures optimal cytoreduction, facilitating the effect of hormonal treatment, but it also accurately specifies myometrial invasion [92]. The hysteroscopic resection technique involves three steps and was first described by Mazzon. The first step consists of the resection of the endometrial lesion, the second step consists of the resection of the surrounding endometrium (approx. 4–5 mm), and the last step consists of the resection of the underlying myometrium (approx. 3–4 mm) [93].

Regarding the duration of hormonal treatment, this is around 6–12 months; after this period and in the absence of tumor regression, radical surgical treatment is recommended [85]. Fertility preservation treatment in patients with endometrial cancer requires careful monitoring of the patients. Most authors recommend an endometrial biopsy once every 3 or 6 months, which can be performed by hysteroscopy or by dilation and curettage [68,88,94,95,96]. The success of the treatment is defined by two negative biopsies at an interval of at least 3 months [90]. Subsequently, the patients receive endometrial biopsies every 3 or 6 months until the moment of obtaining a pregnancy or until the decision of hysterectomy [85].

Several studies have emphasized the importance of using assisted reproduction technologies (ARTs) in patients who had a complete response to hormonal therapy, shortening the time until obtaining a pregnancy and thus minimizing the risk of relapse [94,97,98,99]. Establishing the type of ovarian stimulation and the type of ART protocol is carried out individually for each patient. There are no data to specify the optimal treatment methods and their duration, but it has been suggested that ovarian stimulation with letrozole and gonadotropins is safe [94]. However, young patients with no history of infertility could try to conceive naturally as long as adequate supervision is provided [96].

Regrettably, uterine cancer may be detected at a later stage, rendering these patients ineligible for conservative treatment. In such cases, a hysterectomy, or even more invasive procedures (like pelvic exenterations) become necessary [100,101]. It should be noted that fertility-sparing surgery in advanced endometrial cancer is unconventional and not recommended by guidelines. However, the patients’ desire to become mothers can still be fulfilled with the assistance of assisted reproduction technologies, including surrogate mothers. One of these methods that can be used for patients without partners is represented by pretherapeutic oocyte cryopreservation, with ovarian stimulation protocols currently available for patients with endometrial cancer [102,103,104,105]. Another method that can be used is represented by embryo cryopreservation. This offers the possibility of preimplantation genetic testing, an important aspect for patients with Lynch syndrome who do not want to pass on the genetic mutation to their offspring [106,107].

In vitro maturation (IVM) is a technique used to mature oocytes in the laboratory setting when the oocytes collected are at the germinal vesicle (GV) stage rather than at metaphase II. IVM can be advantageous for patients with polycystic ovaries who are at a higher risk of developing endometrial cancer due to chronic anovulation, as well as those at risk of ovarian hyperstimulation syndrome if subjected to standard ovarian stimulation protocols. Additionally, patients undergoing ovarian tissue freezing may benefit from IVM if GV-stage oocytes are identified during the dissection of the ovarian cortex. Immature oocytes are cultured in specialized media, and if they mature within 24 h, indicated by the extrusion of the first polar body, they can be cryopreserved. Alternatively, if the patient has opted to freeze embryos, the matured oocytes can be inseminated, and the resulting embryos can be cryopreserved at the blastocyst stage [108].

Regarding oocyte cryopreservation, following the process of rewarming, oocytes have achieved survival and fertilization rates surpassing 75%, with live birth rates reaching 35%. Cryopreserved embryos exhibit an exceedingly high survival rate (>95%) upon rewarming, with women under 40 years old having approximately a 40% chance of achieving a live birth in the future. While hundreds of live births have been achieved, the efficiency of the IVM procedure still lags behind standard ovulation induction protocols. Oocytes matured from GVs result in fewer embryos with reduced chances of implantation, pregnancy, and live birth rates compared to conventional IVF.

As extensively discussed in this review, for certain patients diagnosed with endometrial cancer, a hysterectomy is strongly advised. In such cases, utilizing cryopreserved oocytes or embryos remains a viable option to achieve parenthood through the assistance of a gestational surrogate [107] or artificial womb [109], both options surrounded by ethical concerns [110,111]. However, this option is unavailable in numerous countries where gestational surrogacy is explicitly prohibited. Consequently, cross-border reproductive travel has emerged as a recognized phenomenon [112]. Moreover, in some countries, the prohibition of gestational surrogacy has prompted the development of uterine transplants. Several teams, including those in the USA (Texas, Ohio), have reported successful births after uterine transplants. They have effectively navigated the intricacies of these surgeries, particularly when procuring the uterus from living donors rather than deceased donors, while also addressing the associated ethical and moral considerations [113]. There are concerns about the potential fetal adverse effects of a pregnancy brought to term while the mother is under immunosuppressive therapy (used to avoid organ rejection) and finalized by a cesarian section (natural birth not being possible due to the lack of uterine contractility secondary to a lack of innervation of the transplanted uterus). These effects raise several ethical concerns and will not be possible to study until more such pregnancies are finalized and the children resulted are monitored for longer periods of time.

A fertility-sparing surgery algorithm may be applied for selected patients with endometrial cancers (a method not generally recommended for small-cell neuroendocrine tumors, gastric-type adenocarcinoma, or adenoma malignum), depending on whether patients (1) want to preserve their fertility; (2) are medically able to be operated; and (3) have lymphovascular space invasion on cone biopsy. We consider the fertility-sparing algorithm presented in Table 2 as adequate for endometrial cancer. All possible therapeutic options for preserving fertility in gynecologic cancers are summarized in Table 3, together with the possible assisted reproductive technologies (ARTs) which may be employed. After delivering a child, patients who have undergone either a radical trachelectomy or a conization for early-stage endometrial cancer may consider a hysterectomy if they opt to do so, or if they have chronic persistent HPV infection or abnormal Pap tests.

### 4.4. Breast Cancer

According to Globocan statistics from 2022, breast cancer is the second most common cancer, with a total of 2,296,840 cases diagnosed in 2022, and ranks fourth in terms of cancer-related mortality, with 666,103 deaths reported in 2022 according to Globocan reports [34]. It is well established that the incidence of breast cancer rises with age, but there has been an observed 11% increase in new diagnoses among women under 45 years old recently [1]. This shift has led healthcare providers to focus on QoL by modified surgical procedures aimed at limiting side-effects [114,115,116], reconstructive surgery [117], and fertility preservation options for these women, given the long-term toxicity concerns associated with the necessary treatment. Approximately 10% of breast cancer cases are diagnosed in women under 40, indicating that many require onco-fertility care, often disrupting family planning upon diagnosis. Therefore, a multidisciplinary approach is crucial to enable these women to safely carry a pregnancy to term for both them and their babies. Premature ovarian insufficiency is a notable adverse effect of chemotherapy, and long-term hormonotherapy can lead to a diminished ability to carry a pregnancy to term, prompting a review of optimal fertility preservation options for affected women [3].

Among the gonadotoxic chemotherapeutic agents, alkylating agents like cyclophosphamide are noteworthy for their dose-dependent toxicity. The threshold dose of cisplatin for inducing amenorrhea is >7.5 g/m^2^ in women under 20 and >5 g/m^2^ in women over 40 [1]. Managing these agents before treatment is vital as they affect the quality of mature, antral, and pre-antral follicles directly. The ovarian reserve is richer in young women, allowing for subsequent ovarian stimulation techniques or even natural conception in certain cases. Evaluating ovarian reserve through anti-Müllerian hormone levels and antral follicle counting is essential [118]. Another theory suggests that chemotherapy affects the ovarian vasculature, making the ovaries particularly sensitive to toxicity. However, data on newer protocols, possibly less toxic, are still limited. Despite its impact on fertility, chemotherapy significantly influences long-term life expectancy, favoring those who undergo recommended treatment [118]. The gold standard for fertility preservation in young breast cancer patients is oocyte or embryo cryopreservation before commencing chemotherapy, with or without Letrozole and follicle-stimulating hormone. Letrozole ovarian stimulation is considered safe, even for BRCA1 and BRCA2 gene carriers, with or without estrogen receptors, despite temporary estradiol elevation [119]. However, some oncologists still have reservations about this practice, and further research is needed to establish its safety conclusively [3]. In urgent cases, immature oocyte retrieval via laparoscopy can be performed for later use of in vitro maturation. Temporary ovarian suppression using gonadotropin-releasing hormone agonists reduces the risk of premature ovarian insufficiency and increases post-chemotherapy pregnancy success [119]. Limited evidence exists regarding assisted reproductive techniques post-breast cancer treatment, but studies suggest oocyte cryopreservation is associated with fewer recurrences and no negative prognosis [3]. Hormone therapy in breast cancer has no fertility effects, and radiation therapy not involving the pelvis minimally affects oocyte quality. Targeted therapies are safer regarding fertility than chemotherapy or radiation therapy [120].

A prospective study presented in May 2024 [121] enrolling 1213 women with stage 0-III breast cancer who were diagnosed at age <40 and followed for 11 years after diagnosis reported pregnancy attempts and results after diagnosis. A total of 197 participants reported a pregnancy attempt. At least one pregnancy and/or live birth was reported in 73% and 65% of cases, respectively, with the median time from diagnosis to first pregnancy being 48 months. The authors reported that in multivariate analysis, higher age at the time of diagnosis negatively correlated with pregnancy and live births, whereas fertility preservation at diagnosis favored pregnancy. Tumor characteristics, type of cancer treatment, genetic mutations, race, prior history of infertility, and never giving birth before diagnosis did not correlate with lower pregnancy or live birth rates.

### 4.5. Future Directions

Literature data support the safety and efficacy of fertility preservation methods in early stages of gynecologic cancers [122,123,124], but even for more advanced cases, new assisted reproduction techniques can provide tailored options for obtaining a viable pregnancy.

A future direction in onco-fertility is developing uterine transplantation methods, which can offer fertility preservation for cervical, endometrial, and ovarian cancers requiring total hysterectomy. They represent a promising future perspective, but the effects on the fetus of a pregnancy brought to term while being under immunosuppressive medication are still unknown and require additional populational studies. Some of these effects may not be available for study for several decades.

Finally, cryopreserved oocytes and embryos can be used to fulfill the desire for parenthood with the help of a surrogate mother, this being a person whose services are compensated or who can act voluntarily to help a friend or someone in the family [93].

## 5. Conclusions

Advancements in medicine have led to a better understanding and management of gynecological neoplasms. This has resulted in increased survival rates. Once the battle against these neoplasms is won, the issue of preserving the quality of life for these women arises, with fertility preservation being an important aspect of quality of life for women who have not yet fulfilled their family planning desires at the time of diagnosis.

Additionally, it is important for patients to be informed about the available options for fertility preservation and to be encouraged to make informed decisions in collaboration with their medical team. It is worth mentioning that this is not carried out routinely, as evidenced by the articles we have analyzed from the literature as well as from our current practice. It is essential to introduce this branch of onco-fertility into guidelines in order to standardize current medical practice and thus provide all patients with the opportunity for fertility preservation. It is essential to have a multidisciplinary and personalized approach in managing fertility preservation, considering the specifics of each case and the individual desires of patients.

As medical technologies continue to advance, it is expected that fertility preservation options will also evolve to become more accessible and efficient. Despite current challenges and uncertainties, progress in this field promises to offer more hope and improved opportunities for women diagnosed with gynecological cancers regarding the preservation of their ability to have children in the future.

While increasingly integrated into medical practice, fertility preservation remains a source of concern for both specialists and patients alike. Given the existing studies on a limited number of patients and the wide variability in methods used, further randomized controlled trials are necessary to accurately establish the efficacy, safety, and pregnancy rates of these fertility preservation techniques. However, introducing standardized recommendations for onco-fertility into guidelines should be taken into consideration in the future.

## Figures and Tables

**Table 1:** Criteria for fertility preservation in gynecological cancers.

Type of Cancer	Criteria for Fertility Preservation Surgery
Endometrial cancer	▪ Histopathological diagnosis of well-differentiated endometrioid endometrial carcinoma, confirmed by an experienced pathologist; ▪ Tumor is limited to the endometrium on MRI or transvaginal ultrasound examination; ▪ No imaging suspicion of metastasis; ▪ There are no contraindications to the treatment or obtaining a pregnancy; ▪ Low-risk molecular category.
Cervical cancer	▪ FIGO stages IA1-IIA *; ▪ No contraindications to the treatment or obtaining a pregnancy; ▪ There is no imaging suspicion of metastases.
Ovarian cancer	▪ FIGO stages IA-IC (with the possibility of preserving one annex).

* For stages IB2-IIA, neoadjuvant chemotherapy is necessary before conservative surgical treatment.

**Table 2:** Fertility-sparing surgery algorithm for early-stage endometrial cancer.

Stage	Initial Strategy	Evaluation	Ulterior Strategy
Stage IA1	Conization	Negative margins (HSIL or invasive disease) and no LVSI	Surveillance
		Negative margins and LVSI present	Radical trachelectomy and SLN mapping/pelvic lymphadenectomy OR Re-conization and laparoscopic SLN mapping/ lymphadenectomy
		Positive margins	Re-conization (to rule out stage IA2/IB disease) OR Radical trachelectomy and SLN mapping/pelvic lymphadenectomy
Stage IA2	Radical trachelectomy and pelvic lymphadenectomy OR Trachelectomy and SLN mapping OR conization and laparoscopic SLN mapping/pelvic lymphadenectomy	Negative margins and no lymph node involvement	Surveillance
		Positive margins and/or lymph node involvement	Radical non-fertility-sparing surgery OR Chemo-radiotherapy OR re-conization/trachelectomy *
Stage IB1 and selected cases of IB2	Radical trachelectomy and pelvic +/− para-aortic lymphadenectomy	Recommanded for tumors <2 cm Tumors 2–4 cm must be carefully selected for a fertility-sparing approach as they may require postoperative adjuvant therapy due to pathologic risk factors	

* if initial treatment was conization. Note: high-risk molecular categories and histology subtypes should be considered for fertility-sparing surgery on a case-to-case basis, and for such cases, radical non-fertility-sparing surgical options should be recommended.

**Table 3:** Fertility preservation options in gynecological cancers.

Type of Cancer	Stage	Fertility Preservation/ART Option
Cervical cancer	IA1 without LVSI	Conization
	IA1 with LVSI	Radical vaginal trachelectomy/simple trachelectomy + pelvic LND or sentinel node mapping
	IB1	Radical vaginal trachelectomy
	IB2	Abdominal radical trachelectomy/NAC followed by conization or the simple/radical trachelectomy
	IB3	NAC followed by conization or the simple/radical trachelectomy
	IIA	NAC followed by conization or the simple/radical trachelectomy
	Advanced disease	Surrogacy ▪ Ovarian suppression with GnRH before/during chemotherapy; ▪ Ovarian transposition before radiotherapy; ▪ Oocyte cryopreservation before neoadjuvant chemotherapy or combined chemo-radiotherapy; ▪ Ovarian cortex cryopreservation.
Endometrial cancer	IA1	Oral progesterone LNG-IUS Hysteroscopic resection + oral progesterone/LNG-IUS
	Advanced disease	Surrogacy/uterine transplant ▪ Oocyte cryopreservation; ▪ Embryo cryopreservation; ▪ Ovarian cortex cryopreservation.
Ovarian cancer	IA	Unilateral adnexectomy
	IB	Bilateral adnexectomy (with preservation of the uterus and future possibilities of assisted human reproduction)
	IC	▪ Unilateral adnexectomy—if limited to one ovary; ▪ Bilateral adnexectomy (with preservation of the uterus and future possibilities of assisted human reproduction) in bilateral ovarian involvement; ▪ Ovarian transposition before radiotherapy (if cancer is limited to one annex, transposition can be performed for the spared annex if radiotherapy is decided).
	Advanced disease	Surrogacy/uterine transplant ▪ Ovarian suppression with GnRH before/during chemotherapy; ▪ Oocyte cryopreservation before neoadjuvant chemotherapy or combined chemotherapy–radiotherapy; ▪ Ovarian cortex cryopreservation (in case there is an unaffected ovary available for preservation); ▪ Embryo cryopreservation.

ART—assisted reproductive technology; LVSI—lymphovascular space invasion; LND—lymph node dissection; NAC—neoadjuvant chemotherapy; LNG-IUS—levonorgestrel-releasing intrauterine system; GnRH—gonadotropin-releasing hormone.

## Data Availability

The studies reviewed in this manuscript are available online.
